# The relevance of clinical skills in an era of large language models: an adrenal insufficiency case study

**DOI:** 10.5116/ijme.67ce.e5dd

**Published:** 2025-04-08

**Authors:** Fabrizia Faustinella, Hien Nguyen, Winston Liaw

**Affiliations:** 1 Baylor College of Medicine, Department of Family and Community Medicine, Houston, TX, USA; 2 University of Houston Cullen College of Engineering, Department of Electrical and Computer Engineering, Houston, TX, USA; 3 University of Houston Tilman J Fertitta Family College of Medicine, Department of Health Systems and Population Health Sciences, Houston, TX, USA

**Keywords:** Clinical reasoning, Large language models (LLMs), Addison’s disease, Hyperpigmentation, Electronic health records (EHR), Diagnostic errors

## Introduction

Accurate diagnosis in medicine relies heavily on history taking skills and physical examination skills.

Oversights in history-taking and physical examination negatively impact clinical reasoning and can lead to delayed diagnosis, unnecessary tests and treatment, escalating medical costs, and potentially life-threatening consequences for patients.

Artificial intelligence (AI) is emerging as a powerful tool in clinical medicine, revolutionizing various aspects of patient care and medical research. Large language models (LLMs), which are artificial intelligence algorithms that recognize, summarize, translate, predict, and generate language, are being used to facilitate the diagnostic process as clinical decision support tools.

Several factors contribute to the challenges encountered in clinical diagnosis:  adverse systemic factors such as lack of continuity of care, poor communication among health care providers, the use of different EHR systems, increasing patient panels, and a general decline in clinical skills.

We reviewed the case of a 67-year-old man who was seen in our clinic with complaints of abdominal pain, nausea, vomiting, progressively worsening fatigue, weight loss, and back pain for five months.

The patient apparently did not mention skin color changes during any of the previous encounters, and several clinicians didn’t notice the tanning of the skin leading to a much delayed diagnosis of Addison’s disease.

We wanted to determine if a large language model could have expedited such diagnosis based on our patient’s clinical presentation.

## Discussion

Clinical observation and inspection are fundamental to the practice of medicine. Something that is immediately visible when evaluating a patient is the color of the skin which can provide important clues to the underlying medical problem.

Darkening of the skin is present in up to 95% of patients with chronic primary adrenal insufficiency and is considered a hallmark of Addison’s disease.

Our patient’s electronic health record (EHR) showed multiple visits in several outpatient primary clinics, walk-in clinics, and emergency departments, all with similar complaints of abdominal pain, nausea, vomiting, fatigue, and back pain. The patient apparently did not mention the skin color change during any of his medical encounters. Multiple clinicians also failed to notice the tanning of the skin and recognize the abnormal tests that suggested the diagnosis of adrenal insufficiency.

This delay in diagnosis can be explained by several factors which include a lack of continuity of care, poor communication among health care clinicians, disparate EHR systems, and a general decline in clinical skills.^1^^-^^4^

The continuity of clinical information is in large part dependent on the continuity of the physician-patient relationship, but patients often end up seeing, across a multitude of settings, different clinicians, including residents and medical students under the supervision of various attending physicians, and advanced care providers. The fragmentation of care is even greater when different EHRs are used.^5^ Primary care physicians are also more and more pressed for time, with increasing patient panels and shorter office visits, making it more challenging to attentively listen to the patients, carefully review the record, and gather all pertinent information.^6^^-^^10^ Another factor contributing to missed or delayed diagnoses is a general decline in clinical skills such as history-taking skills, physical examination skills, and clinical reasoning skills.^11^^-^^15^

Large language models, which are artificial intelligence algorithms that recognize, summarize, translate, predict, and generate language, are being used to facilitate the diagnostic process as clinical decision support tools.^16^^,^^17^ By querying LLMs or uploading transcripts of conversations, these tools can suggest diagnoses and inform treatment plans. Nevertheless, these tools are limited by the information provided by patients and clinicians.

We started by entering into ChatGPT the prompt “differential diagnosis of abdominal pain, nausea, vomiting, weakness, and back pain.” While ChatGPT generated largely accurate and comprehensive information, Addison's disease was listed as number eight out of eight possible diagnoses, after gastroenteritis, pancreatitis, appendicitis, cholecystitis, nephrolithiasis, cardiovascular causes and diabetic ketoacidosis. When skin color changes were added to the prompt, “differential diagnosis of abdominal pain, nausea, vomiting, weakness, back pain, and hyperpigmentation,” Addison’s disease and adrenal insufficiency appeared as number one diagnosis.

This case shows that if the one key element of the clinical presentation of Addison’s disease, skin color changes, goes unnoticed by the clinician or is not mentioned by the patient during the clinical encounter, the utility of LLMs in making diagnoses may be diminished. In other words, LLMs will not reduce diagnostic errors if key signs and symptoms are missed by the clinician and not mentioned by the patient.

Still, ChatGPT could have been helpful if the skin color changes, although not noticed by the clinician, were mentioned by the patient allowing the algorithm to incorporate it into the history of present illness and taking it into account when generating the differential diagnosis, therefore alerting the clinicians to the diagnostic possibility of Addison’s disease.

The development of multimodal AI tools could have a greater potential to support clinical reasoning especially in complex cases when human oversight might miss critical symptoms.^19^^,^^20^ These advanced  artificial intelligence systems  can analyze and interpret diverse types of medical data simultaneously, like radiologic images (X-rays, MRIs), text from electronic health records, audio recordings (heart sounds),  and wearable sensor readings, providing a more comprehensive picture of a patient's health to improve diagnosis and treatment planning.

## Conclusions

While AI and LLMs hold significant potential in assisting with diagnostic decision-making, their effectiveness is currently limited by the quality and completeness of the input data, i.e. by the information provided by patients and clinicians. Accurate diagnosis in medicine relies heavily on the comprehensive collection of patient symptoms and history.  Both patient self-reporting and physician inquiries are critical in this process. If either party fails to notice or communicate specific symptoms, it can lead to an incomplete or incorrect differential diagnosis. This limitation is also applicable to LLMs used in diagnostic decision-making, as they depend on the data provided to generate accurate outputs.

An AI model cannot infer symptoms or signs that are not explicitly mentioned. In this particular case, the patient and several clinicians did not recognize the significance of the hyperpigmentation, which was crucial to the diagnosis of Addison's disease. Therefore, if the key symptom of hyperpigmentation is not reported, the AI's diagnostic suggestions will be based on incomplete information and won’t be helpful.

Tools that integrate multimodal AI may soon serve as another set of eyes and ears for clinicians. Currently, many LLMs only analyze text, which is influenced by the perspectives of the clinicians and patients. If video, audio, and sensor data were available during encounters, AI could detect abnormalities that were overlooked by the clinician.^19^^,^^20^ Regarding the decline of clinical skills, while a multifactorial phenomenon, it behooves academic medical institutions to examine the causes of such a problem and implement measures to mitigate it.^14^

In the challenging and complex healthcare domain, the use of AI has the potential to improve diagnostics, treatments, and overall patient care.

Research into enhancing data collection methods and integrating good clinical judgment with AI tools can help capturing the complexity of human health and disease, bridging the gap, and ensuring that AI serves as a valuable adjunct to, rather than a replacement for, human clinicians.^20^

### Conflicts of Interest

The authors declare that they have no conflict of interest.
